# Multidimensional Datasets Supporting Vulnerability Assessments of Riverine Bridges in Peru

**DOI:** 10.1038/s41597-025-06374-x

**Published:** 2025-12-13

**Authors:** Alain Jorge Espinoza Vigil, Joel Ccanccapa Puma, Alan Huarca Pulcha, Alejandra Ticona-Quispe, Diego Huarcaya-Perez, Alberth Gonzales-Turpo, Roly Cusihuaman-Casquina, Flor Lucana-Hancco, Julian Booker

**Affiliations:** 1 School of Civil Engineering, Catholic University of Santa Maria, San Jose Urbanization, Yanahuara District, Arequipa, 04013 Peru; 2 School of Civil, Aerospace and Design Engineering, University of Bristol, Bristol, BS8 1TR UK; 3 Postgraduate School, Universidad Continental, Huancayo, 12006 Peru

**Keywords:** Hydrology, Civil engineering, Engineering, Natural hazards

## Abstract

Riverine bridges are critical yet vulnerable infrastructure, constantly exposed to hydrological hazards substantially increased by climate change. Consequently, they require designs that ensure resilience and an adequate useful life cycle. This data descriptor presents data used in two scientific studies focused on assessing the vulnerability of bridges spanning the Chili River in the city of Arequipa, Peru, mainly through hydrological data, terrain and soil data and hydraulic modeling. Therefore, the data employed in both studies enable vulnerability assessments of bridges that encompass both quantitative and qualitative aspects, addressing the data gap in research related to the assessment of riverine bridges. Both studies, by incorporating a diverse range of data types, collectively provide a comprehensive framework for the analysis of riverine bridge vulnerability, rather than functioning as independent investigations. Such data not only serves to assess bridge vulnerability but can also be integrated into future research on infrastructure risk assessments. Proper management of the data presented herein can inform decision-making processes for authorities responsible for implementing resilient and sustainable infrastructure.

## Introduction

Hydrology is the scientific study of the movement, distribution and management of water resources. The impact of climate change on the water cycle includes sudden and accelerated alterations in water behavior and contributes to the emergence of various natural disasters; while a significant part of these events has natural origins, human factors play a crucial role^1^, as effective data management is essential to ensuring the accuracy of hydrological analyses and forecasts^2^. This situation becomes even more relevant due to the great uncertainty and inaccuracy in estimating the effect that climate change may have on the hydrological cycle^3,4^. Therefore, its impact emerges as a determining factor due to the rapid alterations it can cause in hydrological behavior patterns, which considerably increase the risk to the structural integrity of infrastructure^5–7^. This is further exacerbated by the continuous exposure of riverine bridges to extreme hydrometeorological events, positioning the hydrological approach as a key component within vulnerability analyses^8–11^. Consequently, this phenomenon directly affects infrastructure^5^, for instance, as intensity and frequency of floods increase or decrease, it becomes more difficult to assess the risk of failure in a bridge amidst natural disasters^12^. In line with this, Nasr, *et al*.^13^ identified 31 potential risks that could threaten bridge safety. Within these risks, scientific literature agrees that one of the most worrying phenomena is scour, as multiple authors have studied it due to the high probability of failure it can cause in bridge piers^14–16^. Bridges have proven to be vulnerable to natural hazards; therefore, it is important to build and design resilient infrastructure that can endure throughout its anticipated life cycle^6,7^.

Riverine bridges are essential infrastructure in urban environments where watercourses are present, as they constitute a key component of the transportation network. Their construction not only represents a significant investment, but also requires a continuous commitment to their preservation and maintenance^9,17^. In this sense, the vulnerability of bridges to hydrological events demands greater visibility, sensitivity, and rigorous analysis, particularly if the bridge has suffered serious damage or has become completely inoperative. For instance, in 2016 and 2017, the El Niño-Southern Oscillation phenomenon in Peru was responsible for the destruction of 449 bridges^18^. Similar events have been recorded, such as in northeastern India, where intense monsoon rains caused river overflows and water surges that compromised the foundations of riverine bridges, leading to their collapse^19^. Therefore, the conservation and restoration of riverine bridges requires the evaluation of multiple factors, including urban and architectural aspects, environmental conditions, life cycle analysis, seismic activity, as well as social and economic variables^9,10,13^.

It is therefore important to have data that enables the evaluation of future risk scenarios, since, according to Zhang, *et al*.^20^, one of the main causes of bridge failure is the lack of hydrological data to estimate the magnitude of floods, which results in inadequate design. Moreover, topographic data is crucial for modeling generation that simulates both flow and behavior of water^21^, and, additionally, reliable datasets are indispensable for hydrological science and modeling^22^. Risks such as flooding, scour and erosion can be anticipated through correct planning and intervention of infrastructure vulnerable to extreme phenomena. The integration of such information is essential for informed decision-making and correct development of conservation strategies that enhance the resilience of infrastructure in fluvial environments^23^.

With the aim of contributing to the current state of scientific dissemination, a data descriptor is presented based on two previous studies that evaluate the vulnerability of riverine bridges in the environmental, technical, social, and economic dimensions with a database to identify challenges, needs, and improvement actions regarding the resilience of river bridges against floods. This addresses scientific and technical gaps in disaster risk management in high-Andean zones of Peru and can be replicated in major cities worldwide with similar characteristics to critically evaluate the vulnerability of river bridges against maximum floods and a quantitative and qualitative assessment methodology for contemporary, heritage, and historic bridges, using techniques to analyze hydrodynamic forces through 1D and 2D hydraulic models. Specifically, the first study, by Huarca Pulcha, *et al*.^9^, which constitutes Dataset 1, assessed the current condition of eight bridges crossing the Chili River, in Arequipa, Peru, encompassing structures from different historical periods, including heritage bridges (dating back to 1884), republican-era bridges (from the early to mid-20th century), and contemporary structures (from the late 20th century to present). The second study, by Ccanccapa Puma, *et al*.^10^, which constitutes Dataset 2, focused particularly on the Grau Bridge, which crosses the aforementioned river and is one of the most significant heritage bridges in the city of Arequipa. Based on these two studies, a comprehensive dataset that includes background data, hydrological data, terrain and soil data, hydraulic data, and validation data is presented. Although this study does not include a detailed analysis of the deterioration of structural materials due to bridge age, the vulnerability of these structures was assessed through hydrological, hydraulic, and social parameters. This approach is documented by Huarca Pulcha, *et al*.^9^ and Ccanccapa Puma, *et al*.^10^, where vulnerability matrices were developed and applied. These matrices incorporate variables such as flow intensity, scour potential, structural freeboard, proximity to population centers, and socioeconomic vulnerability, providing a comprehensive risk perspective beyond just the effects of structural aging.

**Fig. 1:**
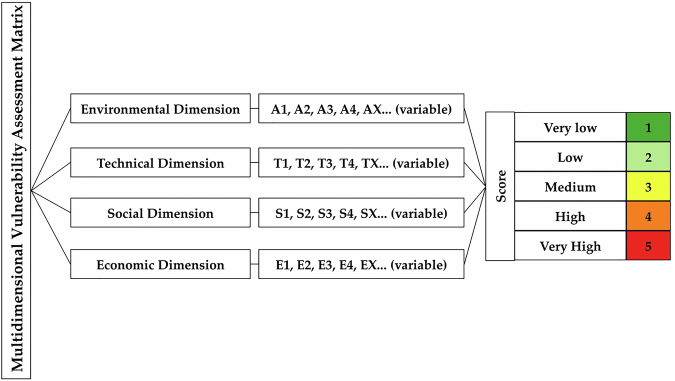
Assessment of vulnerability in river bridges through a multidimensional matrix used in datasets 1 and 2 as a success case in the city of Arequipa, Peru^9,10^.

The objective and significant aspect of this data descriptor is to provide an open and accessible scientific dataset that supports the analysis, evaluation, and management of infrastructure, especially riverine bridges, in response to extreme climatic phenomena such as floods. It is expected that the use and study of the scientific data will contribute to its better understanding and reproducibility, furthermore, this will enable informed decision-making in both academic and civil contexts. The data generated by these methodologies serve as optimal tools based on scientific evidence to guide intervention actions through the multidimensional assessment matrix (Fig. 1). This makes it an optimal tool for prioritizing interventions in river bridges and even more so in the heritage conservation of historic structures, which underscores the need to formulate action measures, promoting a balance between functionality and heritage preservation. Furthermore, the proposed matrix is designed to be replicable and adaptable with more evaluation variables and the use of even more representative hydraulic models such as 3D models, to determine local hydraulic parameters (scour) in computational fluid dynamics (CFD). This study underscores the anticipation of future needs such as the implementation of digital twins (DTs) with accelerometers and sensors to monitor water velocities and levels in real-time during rainy seasons and extreme events, as early warning systems. Also, actors in charge of infrastructure management will benefit from the opportunity to propose new evaluation methodologies based on the supplied data. Hence, data are presented that allow for both qualitative and quantitative assessment of the vulnerability of bridges that suffer from effects of natural disasters.

To further understand the context and current state of the scientific dissemination of data related to hydrology and bridges, and to identify existing research gaps, data descriptors were systematically analyzed. Data descriptors, consisting of formally registered information as an organized dataset, can be rarely found in scientific communities related to hydraulics or civil engineering. Therefore, articles operating datasets were explored with a primary focus on bridges, hydrology, hydraulics, and infrastructure overall. For this purpose, journals indexed in important scientific databases such as Web of Science and Scopus were consulted. Given the observed scarcity of literature related to the proposed topic, previous research with similar characteristics have been collected and organized to address the identified data gap. Table 1 presents key related studies, where the types of infrastructure studied by the authors, as well as their data types, are indicated.

**Table 1:** Compilation of studies presenting both qualitative and quantitative data related to hydrology, hydraulics, or infrastructure vulnerability; the journals in which they are indexed and the repositories where the data can be found are indicated.

Author	Infrastructure	Data type	Journal	Repository
**Indexed journals specialized in data descriptors**				
Loli, *et al*.^24^	Bridges	Bridge data (.xlsx), bridge photographs (jpg), 3D digital models (available in Sketchfab).	Data in Brief	Mendeley Data
Ayzel^23^	Hydraulic infrastructure	Hydrological model outputs and runoff characteristics (.csv), meteorological data (.csv) and Geographic Information Systems (.geojson).	Data	Zenodo
Erpicum, *et al*.^26^	Bridges	Bridges geometry and their location, flood conditions and debris accumulation (.csv).	Scientific Data	Zenodo
Tucker Lima, *et al*.^29^	Hydroelectric dams	Health records, precipitation, dry season length, forest and water cover, river water level, river flow and hydroelectric dams details (.csv and .xlsx).	Scientific Data	Dryad Digital Repository and Amazon Dams Network
**Indexed journals not specialized in data descriptors**				
Ichi, *et al*.^27^	Bridges	Drawings of bridge decks (.dwg), Impact Echo field data (.lvm) and test data (.xlsx), ground penetrating radar data (.csv) and infrared thermography map images of each bridge (.png).	Infrastructures	Scholarly Commons
Loli, *et al*.^25^	Bridges	Diagrams, complementary data reports, images, maps, photographs, data recording tables.	Science of the Total Environment	Not available
Zubieta, *et al*.^28^	Hydraulic infrastructure	Observed rainfall and satellite-based rainfall datasets, hydrometeorological data and hydrological models.	Tecnología y Ciencias del Agua	Not available

Loli *et al*.^24^ developed a rigorous risk assessment framework, validated against an extensive and detailed record of observed damage across 16 bridges in Central Greece. The methodology proposed by Loli *et al*.^25^ addresses the integration of flood hazard intensity index, which incorporates flow velocity and water depth as critical parameters controlling the severity of flood impact on structures, proving decisive for accurate prediction of damage to abutments and foundations. Furthermore, Erpicum *et al*.^26^ addressed another deficiency by creating a unique dataset documenting 71 floating debris accumulations on bridges following the July 2021 floods in Germany and Belgium, systematically detailing the dimensions and heterogeneous composition of accumulations, including anthropogenic objects in addition to floating wood. Regarding structural vulnerability and data for defect detection, Ichi *et al*.^27^ developed a non-destructive evaluation dataset utilizing infrared thermography and ground-penetrating radar to detect subsurface defects, such as delamination, in concrete bridge decks in service. In the realm of hydrology and climate impact, Ayzel^23^ contributed a large-sample dataset of simulated runoff for 425 river basins, filling a clear gap in the representation of river basins, which are of particular interest to local communities. Meanwhile, Zubieta *et al*.^28^ advanced hydrological modeling in sparsely monitored regions such as the Peruvian Andes by evaluating the capability of satellite precipitation products for streamflow simulation, demonstrating similarity with observed rainfall and proving feasible for hydrological modeling. Finally, the Amazon socio-ecological database, compiled by Tucker *et al*.^29^ addresses the deficiency of data dispersion in the Amazon region by integrating multiple sources into an easily accessible resource, providing a broad and reliable foundation for exploring infrastructure development impacts on the complex Amazonian socio-ecological system.

In this study, vulnerability refers to the intrinsic susceptibility of riverine bridges to suffer damage or functional loss when exposed to hydrological hazards. Unlike risk, which represents the combined outcome of a hazard’s intensity and the infrastructure vulnerability. Lamb *et al*.^30^ quantitatively explored uncertainties regarding bridge vulnerability to scour. They consider that failure probability is inherently given by damage caused in the face of flooding, likely due to scour. Such damage is sufficient to cause a safety threat, disruption, and action for infrastructure repair, as it would provoke collapse if left unattended. Pregnolato *et al*.^17^ and Li *et al*.^31^ focus on response at a structural level to identify failure occurrence through an integrated multi-physics modeling framework under hydrological simulations and computational fluid dynamics (CFD) to quantify hydrodynamic forces and structural analysis. This approach seeks to understand vulnerability not only in the foundation (scour) but also in the superstructure (bridge) due to hydrodynamic forces during flooding. Tubaldi *et al*.^32^ point out that bridge type characteristics constitute the most vulnerable component, such as the deck and abutments. They promote the urgency of developing models that consider cumulative risk effects (less intense but more frequent events) instead of focusing on a single design return period (a non-physical measure) that fails to capture event duration (crucial for scour). This leads to the need to identify hazard intensity measures, combining flow depth/velocity^10,33^ and scour depth to describe the joint effect of flood actions on bridge vulnerability.

Due to the unavailability of data in some of the studies presented, it was not possible to specify the file format of the information. The presented data descriptor is connected to these studies by proposing an innovative approach that merges open data and geospatial analysis, seeking not only to understand the state of infrastructure, but also to foresee future needs. It is important to emphasize that the data descriptors presented in Table 1 are a clear basis for future research, especially in the engineering context. Although the results are crucial in research, it is equally important to have the data collected and freely accessible. However, it was observed that the number of articles providing transparency of their results through datasets is very limited, restraining the reproducibility of the methodologies to corroborate that the data are reliable and can be used in different contexts^27,29^. This article contributes by bridging the gap between the assessment and management of riverine and heritage bridges and the technical data to foresee future risks.

The studies summarized in Table 1 provided the initial foundation for the development of the datasets presented in this work. Specifically, they supplied hydrological and structural insights that guided the selection of input data sources, the design of the field campaigns, and the subsequent integration of hydrological and topographical information related to the Chili River and its bridges. Building on these precedents, our dataset advances the state of the art by offering, for the first time in this region, an openly accessible and systematically curated repository that integrates hydrological records, topographic surveys, structural bridge assessments, and photographic documentation. Unlike previous studies, which focused on isolated components, this dataset combines both quantitative (hydrological and topographical measurements) and qualitative (structural and historical evidence) data in standardized formats directly compatible with modeling tools such as HEC-HMS and HEC-RAS. In doing so, it addresses a critical gap in the dissemination of bridge-related hydrological information in Andean river systems, providing researchers and practitioners with a comprehensive resource to improve flood risk analysis, vulnerability assessment, and infrastructure resilience in developing regions.

## Methods

### Data collection

**Fig. 2:**
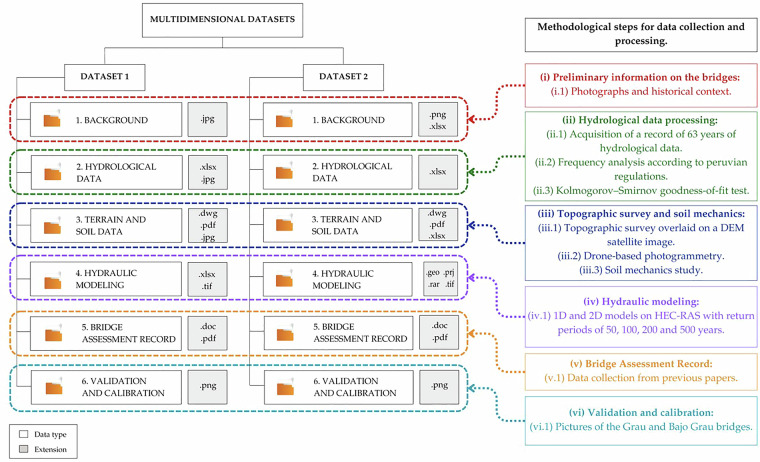
Organizational diagram of Datasets 1 and 2, showing the folders in which they were classified and the file extensions of the data. The methodological steps and their connection to the files are also displayed.

Figure 2, in addition to showing the distribution of both datasets and the file extensions contained within these folders, illustrates the methodological steps followed by the authors of datasets 1 and 2. Both datasets share several data collection and processing methods; this is illustratively explained in Fig. 1, which directly links the methods used to the type of information collected.

### Study area

**Fig. 3:**
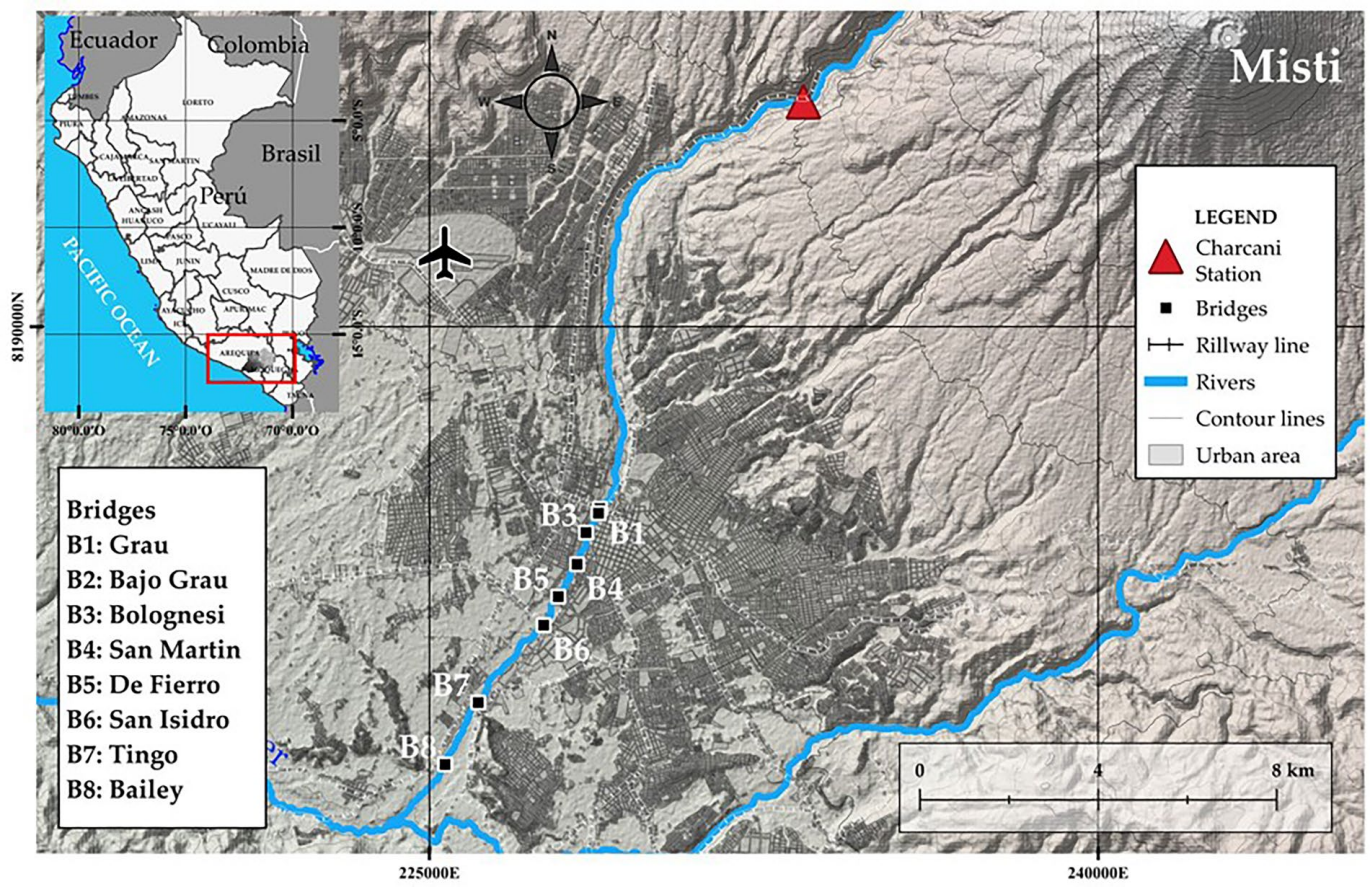
Bridge’s location spanning the Chili River in the city of Arequipa y Peru.

The study area is located in Arequipa, a city in southern Peru, situated at an approximate elevation of 2,300 meters above sea level, with a population of over 1.3 million inhabitants. The presented dataset is related to the analysis of eight bridges located along the Chili River, which lies within the Quilca – Chili basin, part of the Pacific Ocean watershed^34^. This basin covers an area of 13 457.01 km², and the hydrographic unit where the Chili River is situated, called Middle Quilca – Vitor – Chili, covers a total of 2347.24 km^2^^35^. The Chili River extends for 88.2 km; however, the presented data focuses only on bridges spanning a 6.95 km segment. Moreover, the flow of this water resource is regulated by the Aguada Blanca dam, which is part of the Salinas and Aguada Blanca National Reserve, and is of critical importance as it serves as the main water source for the city of Arequipa^36^. It is important to note that, beyond these technical aspects, the study of this river section is of great interest because, in the past, bridge closures were necessary due to imminent risks caused by the sudden rise in water levels of the Chili River (Fig. 3).

To analyze the vulnerability of riverine bridges, (i) preliminary information on the bridges was collected, based on (i.1) photographs and historical context. This allowed for a general understanding of the condition of the bridges, as this information verifies the age of the structures and their conservation status. Subsequently, the study continued with the (ii) hydrological data processing, necessary to analyze the precipitation in the study area. For this, (ii.1) a 63-year hydrological data record from the Peruvian entity AUTODEMA^37^ was acquired. Additionally, according to the Manual of Hydrology, Hydraulics and Drainage of the Peruvian Ministry of Transport and Communications^38^, (ii.2) a statistical analysis was conducted to determine precipitation, intensities, and maximum discharge distributions. Then, following the recommendations of the aforementioned manual, the (ii.3) Kolmogorov–Smirnov test was carried out, allowing the verification of the goodness-of-fit of the distributions. Regarding the conditions of the study area, it was necessary to identify the soil properties to obtain roughness coefficients. For this purpose, (iii) a topographic survey and a soil mechanics study were conducted. For Dataset 1, (iii.1) a topographic survey from the Peruvian agency, the National Water Authority^34^, was overlaid on a DEM satellite image from ALOS PALSAR, whereas for Dataset 2, (iii.2) photogrammetry supported by a DJI Phantom 4RTK drone was performed. To determine soil properties, a (iii.3) soil mechanics study was carried out through test pits 1.5 m deep. With all the data collected and processed in the previous steps, it was possible to perform the (iv) hydraulic modeling. This consisted of (iv.1) 1D and 2D models using HEC-RAS (simulation software) with return periods of 50, 100, 200, and 500 years. Additionally, (v) a bridge assessment record is presented through (v.1) the data collection from the previous research conducted by Huarca Pulcha, *et al*.^9^ and Ccanccapa Puma, *et al*.^10^. Finally, for Dataset 2, (vi) a validation and calibration stage was carried out through (vi.1) the collection of photographs of the Grau and Bajo Grau bridges during the rainy season in the city of Arequipa.

HEC-RAS (Hydrologic Engineering Center – River Analysis System) is a hydraulic model that solves the Saint-Venant equations (or shallow water equations) in 1D and 2D to describe flow in natural and artificial channels^39^. In 1D mode, the river is represented as a succession of cross-sections connected by the channel axis. Flow is considered one-dimensional in the main direction. The equations it solves are the 1D Saint-Venant equations, which are Eq. (1) for continuity or conservation of mass and Eq. (2) for momentum or conservation of momentum.

1 $$\frac{\partial A}{\partial t}+\frac{\partial Q}{\partial x}={q}_{l}$$

Where: A is the flow area (m²), Q is the discharge (m³/s), and q is the lateral discharge per unit length (inflow or losses) in (m²/s).

2 $$\frac{\partial Q}{\partial t}+\frac{\partial }{\partial x}\left(\frac{{Q}^{2}}{A}\right)+{gA}\frac{\partial h}{\partial x}+{gA}\left({S}_{f}+{S}_{0}\right)=0$$

Where: h is the water depth (m), g is gravity (m/s²), ${S}_{0}$ is the bed slope, and ${S}_{f}$ is the friction slope.

Under gradually varying flow conditions and steady regime, HEC-RAS also applies Eq. (3) for energy, known as Bernoulli’s equation:

3 $$E=z+y+\alpha \frac{{V}^{2}}{2g}$$

Where: z is the bed elevation, y is the water depth, and α is the kinetic energy coefficient.

**Fig. 4:**
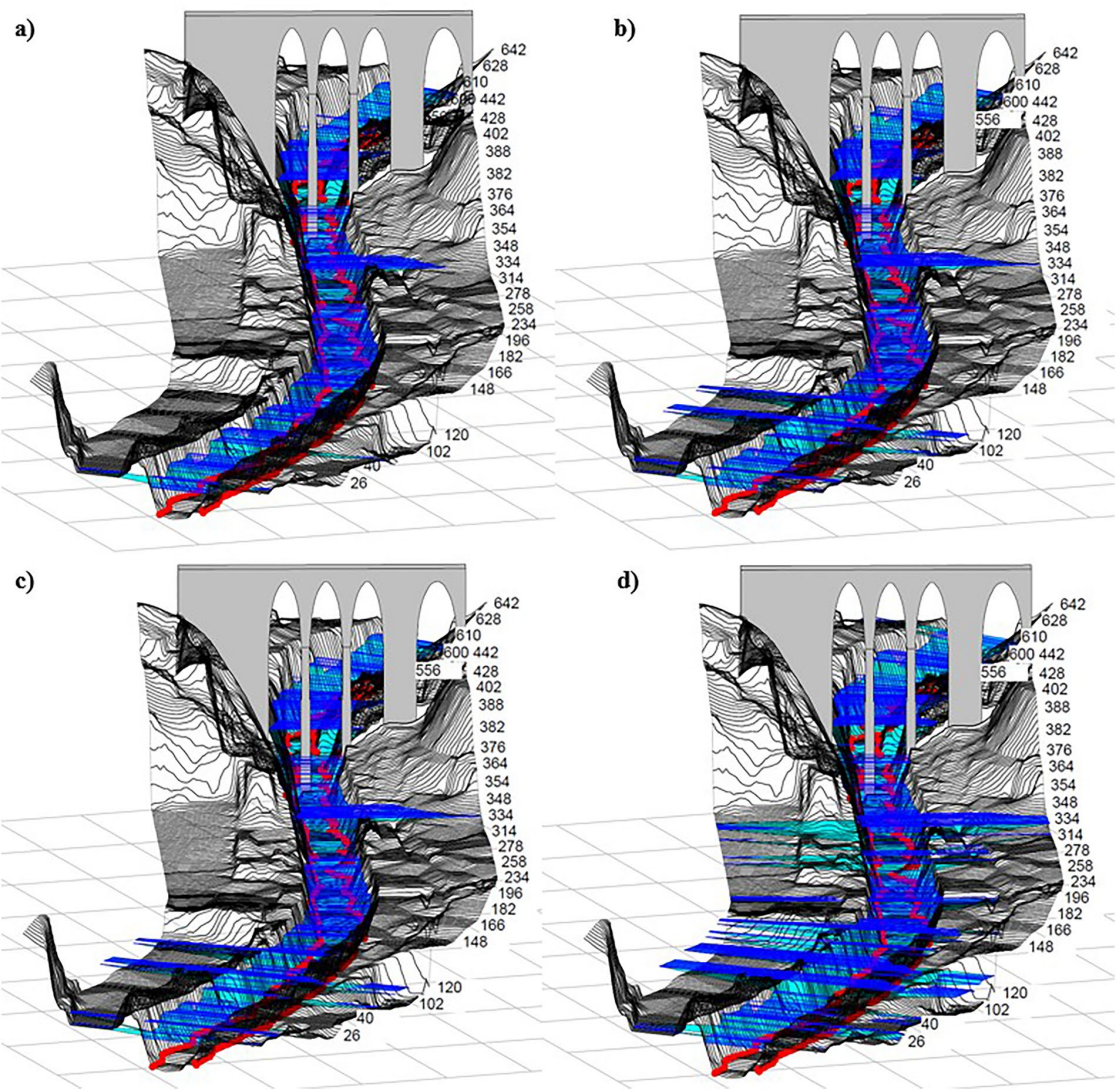
1D modeling of the Grau Bridge^10^ in HEC-RAS (Data set 2) for different flow rates: (**a**) T50 = 223.0 m³/s; (**b**) T100 = 248.5 m³/s; (**c**) T200 = 273.0 m³/s; y (**d**) T500 = 304.3 m³/s.

An example is the numerical simulation of article 2 (Fig. 4) of data set 2.

In the 2D model, the flow domain is discretized into a mesh (triangular or quadrangular) and water is allowed to move in two horizontal directions (x, y).

The equations it solves are the 2D Saint-Venant equations: Eq. (4) for continuity and Eqs. (5) and (6) for momentum in the x and y directions.

4 $$\frac{\partial h}{\partial t}+\frac{\partial ({uh})}{\partial x}+\frac{\partial (\upsilon h)}{\partial y}=q$$

Where: h is the depth (m), $u,\upsilon$ are the velocities in the x and y directions (m/s), and q is the water source or sink (m/s).

5 $$\frac{\partial ({uh})}{\partial t}+\frac{\partial }{\partial x}({{uh}}^{2}+1/2g{h}^{2})+\frac{\partial (u\upsilon h)}{\partial y}=-{gh}\frac{\partial z}{\partial x}-\frac{{\tau }_{{bx}}}{\rho }$$

6 $$\frac{\partial (\upsilon h)}{\partial t}+\frac{\partial }{\partial y}({\upsilon h}^{2}+1/2g{h}^{2})+\frac{\partial (u\upsilon h)}{\partial x}=-{gh}\frac{\partial z}{\partial y}-\frac{{\tau }_{{by}}}{\rho }$$

Where: z is the bed elevation (m), $\rho$ is the water density, and ${\tau }_{{bx}},\,{\tau }_{{by}}$ are the bed shear stresses in x and y directions.

**Fig. 5:**
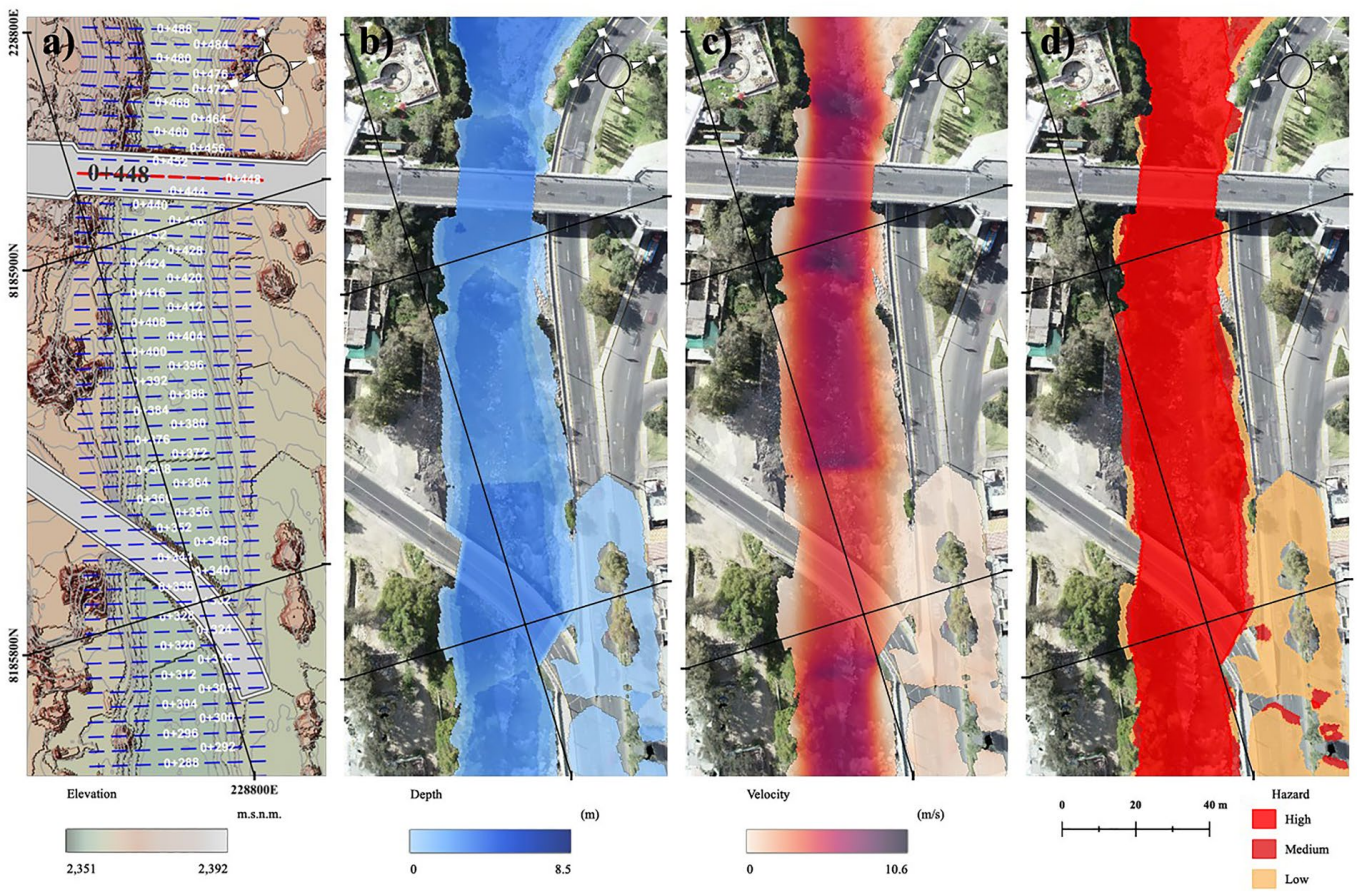
2D modeling of the channel where the Grau bridge^10^ is located in HEC-RAS (dataset 2) for a return period of T = 200 years (273.0 m³/s). (**a**) digital terrain model (DTM) with cross-sections; (**b**) maximum depth map (m); (**c**) maximum velocity map (m/s); and (**d**) hazard map derived from maximum depths and velocities.

An example is the numerical simulation of article 2 (Fig. 5) of data set 2.

Table 2 shows a summary of the characteristics of the 1D/2D models used for the analysis of articles 1 and 2 (datasets 1 and 2).

**Table 2:** Modeling characteristics of 1D and 2D in HEC-RAS for datasets 1 and 2.

Characteristics	HEC-RAS 1D	HEC-RAS 2D	Data set 1 (1D)	Data set 2 (1D)	Data set 2 (2D)
**Mathematical formulation**	1D Saint-Venant equations (continuity and momentum).	2D Saint-Venant equations (shallow water equations).	Continuity and momentum	Continuity and momentum	SWE-EM (Stricter Momentum)
**Main dependent variable**	Discharge Q(x,t) and water depth h(x,t).	Water depth h(x,y,t) and velocities $u$ (x,y,t), $\upsilon$ (x,y,t)	Steady flow with discharges of T = 100 (247.5 m³/s), T = 140 (259.3), T = 200 (271.5 m³/s) and T = 500 (301.9 m³/s) years.	Steady flow with discharges of T = 50 (223 m³/s), T = 100 (248.5 m³/s), T = 200 (273 m³/s) and T = 500 (304.3 m³/s) years	Unsteady flow for discharges of T = 50, T = 100, T = 200, and T = 500 years
**Flow direction**	Flow restricted to the longitudinal direction of the channel.	Free flow in two horizontal directions (x,y).	135 cross-sections	321 cross-sections perpendicular to the channel axis	—
**Geometric representation**	River profile through cross-sections perpendicular to the channel axis.	2D mesh that discretizes the entire floodplain.	—	—	9503 cells Spacing DX = 5 m Spacing DY = 5 m
**Represented processes**	Water levels and discharges along the channel axis. Handles structures (bridges).	Spatial flow dynamics: overflows, recirculation zones, floodplain flows.	Contraction coefficient (0.3), expansion (0.5), dredging (Cd = 1.33), and semicircular pier shape (K = 0.9)	Contraction coefficient (0.3), expansion (0.5), dredging (Cd = 1.33), and semicircular pier shape (K = 0.9)	Overflows, recirculation zones, floodplain flows
**Boundary conditions**	Defined at upstream and downstream cross-sections.	Defined at the upstream and downstream mesh boundaries.	Mixed flow (Normal Depth) for subcritical and supercritical flow	Mixed flow (Normal Depth) for subcritical and supercritical flow	Upstream and downstream (BClines)
**Numerical resolution**	Implicit finite difference scheme at discrete sections.	Finite volume method with conservative flow solution in cells.	Finite difference method (FDM)	Finite difference method (FDM)	Finite volume method (FVM)
**Computational requirements**	Low memory and computational time requirements.	High computational demand (depends on mesh size and refinement).	AMD Ryzen 7 5800H with Radeon Graphics	13th Gen Intel(R) Core (TM) i7-13650HX	AMD Ryzen 7 5800H with Radeon Graphics
**Limitations**	Does not adequately represent lateral flows, recirculations, or complex overflows.	High demand for topographic data and simulation times.	Discrete cross-section, linear, simplified floodplain	Discrete cross-section, linear, simplified floodplain	DTM (DJI Phantom 4 RTK drone, every meter) and time step based on Courant criterion (1 s)

## Data Records

The data are available at the Mendeley Data repository (Version 4): (10.17632/bnc9r2vwj9.4)^40^. The dataset is described in two folders, as displayed in Fig. 2. Datasets 1 and 2 contain key information for the development of studies related to hydrological flow, topography, hydraulic modeling and other aspects essential for the vulnerability assessment of riverine bridges. These datasets include various file types (.jpg, .xlsx, .dwg, among others) that support each stage of the analysis, starting from background collection to model validation and calibration.

### Dataset 1

The data provided are distributed in folders organized as follows:

#### Background

Described in nine folders, eight of the folders contain photographs from 2022, taken personally by the authors, in.jpg format and distributed according to the eight bridges analyzed (1. Grau Bridge; 2. Bajo Grau Bridge; 3. Bolognesi Bridge; 4. San Martin Bridge; 5. Bolivar (Fierro) Bridge; 6. San Isidro Bridge; 7. Tingo Bridge; 8. Bailey Bridge). The ninth folder (9. Google Earth) contains a compilation of satellite photographs adapted from Google Earth of each bridge. These photographs were adapted from Google Earth^41^.

#### Hydrological data

##### Chili river flow register

It presents a file in .xlsx format under the name of “2.1.1 Maximum flows”, which lists the Chili River flow information according to both the Peruvian entity AUTODEMA^37^ (2005–2022) and to Espinoza Vigil and Booker^5^ (1960–2004).

Another file in the same format under the name of “2.1.2 Statistical aspects” presents the following sheets: “Statistical Analysis”, defines both maximum and minimum statistical parameters and values; “Outlier Data Test”, corroborates the veracity of the values to be analyzed through the three-parameter gamma distribution; and sheet 3, “Return Period”, which, based on the Manual of Hydrology, Hydraulics and Drainage of the Peruvian Ministry of Transport and Communications^38^, makes it possible to deduce the flows of the Chili River with a return period of 100, 140, 200, 500 and 1000 years, employing the Hydrognomon and Hidroesta 2.0 software, both used to validate the statistical analysis.

##### Flows considering surrounding streams

The “2.2.1 Flows for Modeling” folder contains the representation of the surrounding streams El Pato, San Lazaro, Venezuela and Los Incas as a.jpg file under the name of “Distribution model”. Also, flow rates are analyzed and determined for modeling in the .xlsx file “Flows for modeling”.

The “2.2.2 HEC-HMS” folder presents modeling files for return periods of 100, 200 and 500 years, considering the influence of neighboring torrents studied by Ramos Fernández and Saldivar Condori^42^.

#### Terrain and soil data

##### Topography

This folder presents the combination of a topographic survey provided by the Peruvian National Water Authority^34^ and a Digital Elevation Model (DEM) satellite image from ALOS PALSAR (Advanced Land Observing Satellite Phased Array type L-band Synthetic Aperture Radar), a radar instrument for earth observation. The data corresponds to the marginal strip of the Chili River, previously processed as a topographic surface raster of the channel, available in both.dwg and.pdf formats.

##### Plan and profile views

This folder presents the visualization of the plan and profile view of the bridges, detailing data on cross sections. Additionally, dimensions are presented in plans, described by elevation and horizontal measurements. The schematic representation of the Baily bridge infrastructure is included, along with the cross-sectional views of the remaining seven bridges (.dwg), studied by Concha Zeballos and Miranda Vega^43^, which were validated on site.

#### Hydraulic modeling

##### Roughness coefficient and bridge cross-sections

Condenses information from the eight bridges used in the HEC-RAS software in a .xlsx file to calculate roughness coefficients for HEC-RAS and flow conditions requested by the software to find the progressives near each bridge.

##### HEC-RAS

The folders “1. Project”, “2. Projection” and “3. Terrain” show the information provided by the HEC-RAS software after uploading the data for modeling. In addition, the “Combination” file is shown in.tif format.

#### Bridge assessment record

This folder contains assessments of the eight bridges under study in both.doc and.pdf formats. These were obtained from research conducted by Huarca Pulcha, *et al*.^9^

#### Validation and calibration

Calibration and validation were performed considering a 5-year return period, since the reservoir is regulated by AUTODEMA, which typically releases flows of approximately 100 m³/s during intense rainfall events in the upper catchments^33^. Evidenced by audiovisual material in folder 6. validation and calibration.

### Dataset 2

The data provided are distributed in folders organized as follows:

#### Background

##### Photographic evidence

Photographs of the Grau Bridge in 1889 and 1930 are presented, in addition to its location in the middle watershed Quilca - Chili in.png format.

##### History

Timeline data of the Grau Bridge construction over time, from 1865 to 1890, in both.png and .xlsx format.

##### Grau and Bajo Grau bridges

It contains two folders: one with a representative image of the Grau Bridge and the other with an image of the Bajo Grau Bridge, both in.png format.

#### Hydrological data

This folder contains, in the “Maximum Flows” file, the maximum flow data records obtained from AUTODEMA^37^ and from the Charcani hydrological station with a record of 63 years (1960–2022); also, their statistical analysis is shown in the “Frequency Analysis” file. Additionally, evaluation of outlier data of the flow records was carried out through a non-parametric evaluation in the “Outliers Analysis” file. All of these files are displayed in .xlsx format.

#### Terrain and soil data

##### Topographic survey

This folder contains the topographic survey of the Chili riverbed where the infrastructure is situated. Contour lines are presented in.pdf and.dwg format. Additionally, a.rar file named “DEM – Grau Bridge”, provides a three-dimensional representation of the structure and its surrounding topography. This allows for an accurate visualization of the terrain elevation and structural height.

##### Architectural drawing

This folder contains the architectural plan and structural details of the Grau Bridge in.pdf and.dwg format.

##### Soil data

This folder contains results from the determination of mean particle size (d_50_) through sieve granulometric analysis following the Peruvian Technical Standard NTP 339.128^44^. The file is in .xlsx format.

#### Hydraulic modeling

##### Unidimensional modeling (1D)

This folder contains the representation of the digital terrain model through sections every five meters of the riverbed where the infrastructure is located. The file is in.geo format.

##### Bidimensional modeling (2D)

This folder contains the representation of the digital terrain model through a pixel structure arranged in rows and columns with elevation information in.tif format. These files are compressed in a .rar file.

#### Bridge Assessment Record

This folder contains the assessment of the Grau Bridge in both.doc and.pdf formats. This was obtained from research conducted by Ccanccapa Puma, *et al*.^10^

#### Validation and calibration

##### Grau bridge

Photographs of the Chili riverbed, focusing mainly on the Grau Bridge during the rainy season, are provided in.png format to support and validate the results.

##### Bajo grau bridge

Photographs of the continuation of the Chili River’s course during the rainy season, featuring a view of the Bajo Grau Bridge. These images, provided in .png format, contribute to demonstrating and corroborating the results.

## Technical Validation

For the topographic component of Dataset 1, the corresponding database was formally requested from the National Water Authority^34^, which is the highest technical and regulatory authority of the National Water Resources Management System in Peru. However, as the surrounding area of the Chili River strip was not verified, the topographic survey was overlaid on a 12.5-meter resolution satellite image, thereby enhancing the accuracy of the hydraulic modeling. This information was subsequently validated using tools such as satellite images, altimeters, and echo sounders to ensure greater accuracy in the elevation and terrain representation. Whereas the drone-based topographic survey of Dataset 2 was personally elaborated by the authors with a DJI Phantom 4RTK drone. The device weighs 1388 g and is equipped with a 4 K video camera capable of capturing images with a resolution of 20 MP. Additionally, it features an obstacle detection system in five directions and a sensor that enhances the precision of the details in the captured images. Regarding measurement errors, variations are observed in both horizontal (X and Y) and altitude (Z) coordinates (Onchi-Ramos, *et al*.^45^). The planimetric error, which refers to the error in horizontal coordinates, ranges from centimeters to decimeters, depending on the operational conditions. In order to obtain a more reliable simulation of the interaction between the Chili River and the bridge under analysis, this survey was established using planimetric and altitudinal controls, consisting of geodetic points and topographic control points (benchmarks) for georeferencing. The quality of the survey was verified in accordance with the guidelines outlined in the Peruvian Geodetic Technical Standard^46^.

The hydrological data, as detailed in the Data Records section, were requested from the Majes Autonomous Authority (AUTODEMA^37^), a body of the Regional Government of Arequipa responsible for managing the Majes Siguas Special Project, an initiative aimed at water resource management for irrigation, energy generation, and the socioeconomic development of the region. These governmental institutions are responsible for providing validated information on water resources for both public and private infrastructure projects in Peru. They perform quality control on the recording of meteorological and hydrological data, as they go through two levels: the first one consists of identifying doubtful values by assessing their adherence to physical and climatological limits, as well as detecting internal and spatial inconsistencies. The second level involves the analysis of the questionable data, ensuring its verification and validation to maintain data integrity.

Specifically, Dataset 1 confirmed and validated the statistical analysis of the hydrological data using the Hidroesta 2.0 and Hydrognomon software. Both programs were employed to determine the lowest theoretical delta (maximum distance between distributions) based on the Kolmogorov-Smirnov test, which facilitated the selection of the most appropriate distribution parameters.

Whereas Dataset 2, although specific to the Grau Bridge, presents a soil mechanics study that required the excavation of a test pit 1.5 meters deep, enabling the extraction of the necessary sample for laboratory analysis and a granulometric test. These tests were conducted in accordance with the specifications outlined in the Peruvian Technical Standard NTP 339.128^46^, which, as indicated in the same document, has as its antecedent the ASTM D422-63, Standard Test Method for Particle-Size Analysis of Soils, which describes the procedures to determine the particle size distribution of soils by sieving and sedimentation methods.

## Usage Notes

### Dataset 1

#### Background

With this photographic evidence it is possible to see the low water level of the Chili River through the bridges studied. The environmental conditions, the state of the infrastructure in a qualitative manner and the levels of water marks from previous periods should be observed.

#### Hydrological data

Files of type .xlsx and HEC-HMS files are presented, as well as the distribution model in.jpg format to visualise the amount of flow that discharges directly into the river for each bridge. A statistical analysis was carried out in Hydrognomon and Hidroesta 2.0 software to find the most appropriate relationship and, in turn, to find files processed in HEC-HMS that take into account neighbouring torrents to obtain the hydraulic modeling flow.

#### Terrain and soil data

These contour lines can be processed using Civil 3D software to export them in.tif format, which can then be used in the HEC-RAS software for hydraulic modeling. The “Plan and Profile Vies” folder provides files in.dwg format and are also available in.pdf format for a quick visualization of each studied bridge.

#### Hydraulic modeling

The data presented is used to evaluate the clearance height and flooding parameters and to determine the extraordinary maximum water levels, which were used in the bridge vulnerability analysis.

#### Bridge assessment record

These data can be used to evaluate the qualitative and quantitative aspects of the eight bridges under study. It also provides multiple parameters that ensure the inclusion of social, environmental, technical, and economic dimensions.

#### Validation and calibration

The data is organized into nine subfolders: eight of them correspond to photographic records for each of the bridges analyzed (1. Grau Bridge; 2. Bajo Grau Bridge; 3. Bolognesi Bridge; 4. San Martín Bridge; 5. Bolívar (Fierro) Bridge; 6. San Isidro Bridge; 7. Tingo Bridge; 8. Bailey Bridge). These folders contain images in.jpg format captured during field visits and inspections, showing hydrological traces, water levels, and flow conditions observed during high-flow events, which serve as qualitative validation of the simulated results.

The ninth folder includes a single file in.pdf format titled “Calibration and Validation Report”, which summarizes the procedures, criteria, and parameters used to calibrate the HEC-RAS hydraulic model and to validate the hydrological conditions. The document details the comparison between simulated water levels and observed conditions, referencing photographs and measurements taken *in situ* to ensure the reliability of the modeling framework.

### Dataset 2

#### Background

The photographic evidence and historical data contain relevant facts about the foundation of the Grau Bridge, historical events leading to its construction, and its inauguration in 1888.

#### Hydrological data

These records undergo an outlier data evaluation to identify atypical data. In the same way a frequency analysis to determine the theoretical probability distribution function that best fits the maximum flow data series, is carried out using a non-parametric Smirnov-Kolmogorov test with the support of Hydrognomon v4 software.

#### Terrain and soil data

This folder contains the terrain contour lines, presented in.dwg format at one-meter intervals, obtained from a topographic survey conducted with a drone over the Chili River channel to create the digital terrain model. These data were used as input for one-dimensional (1D) and two-dimensional (2D) hydraulic numerical models. The folder also contains the architectural plan of the Grau Bridge in.dwg format that can be used to determine the geometric dimensions for subsequent representation and simulation in HEC-RAS software.

#### Hydraulic modeling

It contains the digital terrain representation by sections in.geo format, previously extracted from the compressed.rar file. This dataset was prepared for the two-dimensional simulation of the Chili River channel, using cross sections of 650 meters, which can be employed to develop the HEC-RAS hydraulic model.

#### Bridge assessment record

These data can be used to evaluate the qualitative and quantitative aspects of the Grau Bridge. It also provides multiple parameters that ensure the inclusion of environmental, technical, social and economic parameters.

#### Validation and calibration

**Fig. 6:**
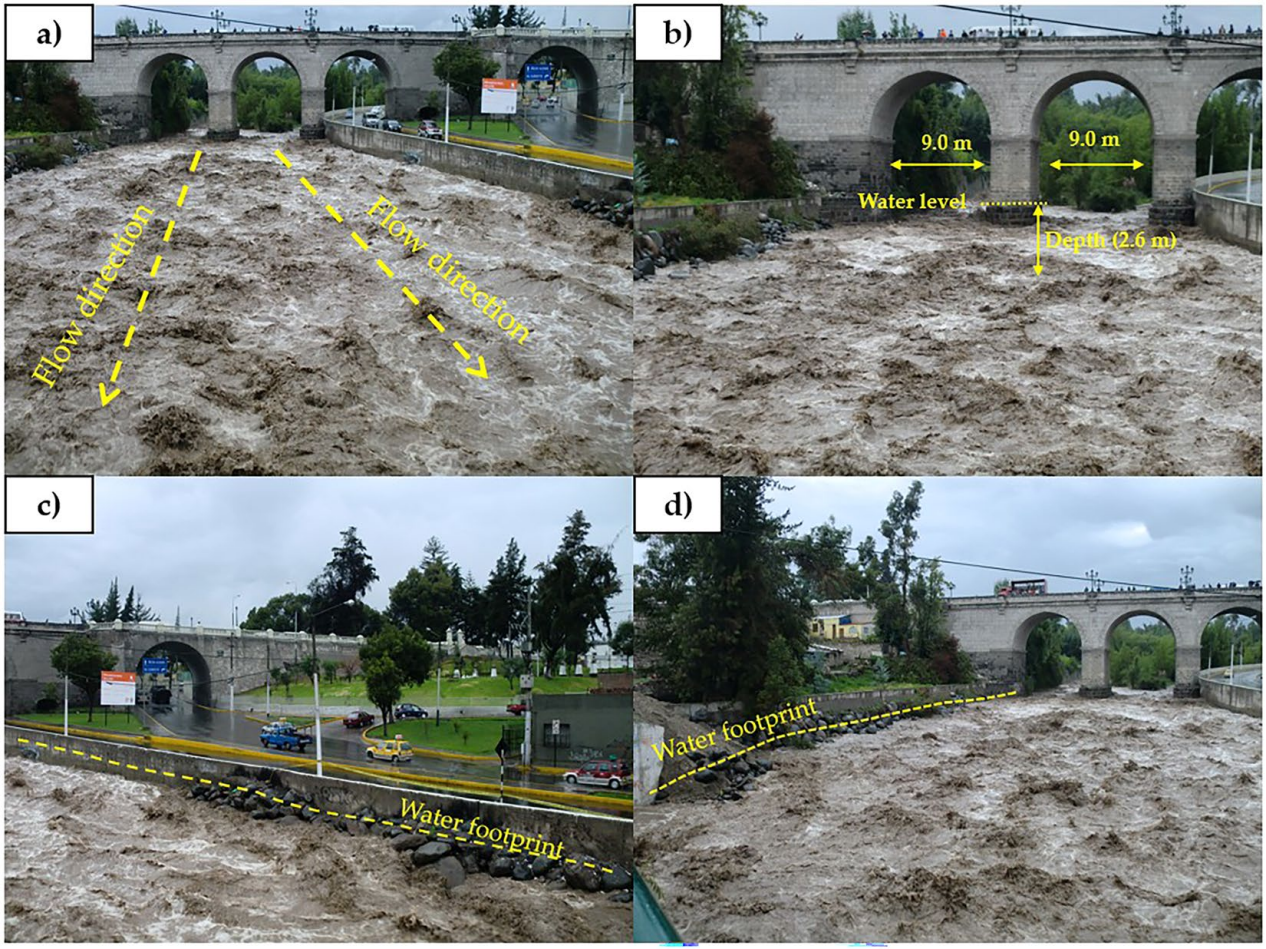
Historical event of 2012 in the Chili River, (**a**) flow direction for the analysis of the Grau bridge, (**b**) water level and depth representing a discharge of 125.5 m³/s at the Grau bridge, (**c**) and (**d**) hydrological traces left by intense rainfall in the upper part of the Quilca-Chili basin, which occur approximately every 5 years in the city of Arequipa.

The calibration of the Chili River consisted of determining water levels, i.e., hydrological traces left by historical events, such as the year 2012 (Fig. 6), where as a result of intense rainfall in the upper part of the Quilca-Chili basin, the dam system operated by AUTODEMA began to release excess water flow from the spillways, increasing the discharge of the Chili River and approximately every 5 years triggering floods^33^. This discharge of 125.5 m^3^/s is consistent with the frequency analysis presented by the Charcani station and represents a water depth of 2.6 m at the Grau bridge pier (Fig. 6b). In this manner, calibration was performed with the HEC-RAS 1D hydraulic model for a return period of T = 100 years, validating the discharges for return periods of T = 100, 200, and 500 years. Photographs are provided to evidence the results obtained through the analysis of quantitative and qualitative data.

## Data Availability

The dataset is available at 10.17632/bnc9r2vwj9.4.

## Code availability

No custom code has been used in this study.
